# Influence of 3D Printing Parameters on the Mechanical Properties for Numerical Simulation of the Poly (Lactic Acid)

**DOI:** 10.3390/polym17172271

**Published:** 2025-08-22

**Authors:** Dorin-Ioan Catana, Denisa-Iulia Brus, Mihai-Alin Pop, Mihai-Alexandru Luca

**Affiliations:** 1 Department of Materials Engineering and Welding, Transilvania University of Brasov, 500036 Brasov, Romania; luca.mihai@unitbv.ro; 2 Interdisciplinary Doctoral School, Transilvania University of Brasov, 500036 Brasov, Romania; denisa.brus@unitbv.ro; 3 Department of Materials Science, Transilvania University of Brasov, 500036 Brasov, Romania; mihai.pop@unitbv.ro

**Keywords:** additive manufacturing, poly (lactic acid), mechanical properties, printing parameters, numerical simulation

## Abstract

Additive manufacturing is a relatively new and modern technological process that is still under development and progress, with a wide range of materials. This study presents the evolution of the mechanical properties of poly (lactic acid) depending on the main parameters of additive manufacturing: speed, infill density, and temperature. In this study, the range considered for each parameter is the one commonly used in printing activities. The study shows that within the analysed range, the relationship between the property values and printing temperature is expressed through third-degree polynomial equations, regardless of the infill density. This study also presents the evolution of the properties as a function of two printing parameters, specifically temperature and infill density. Through statistical analysis, equations were determined that describe the relationship between the studied properties and the printing parameters. The existence of these equations facilitates their integration into commonly used simulation programs for design. Additionally, this study presents the results of the simulation process for tensile tests on additively manufactured structures, based on data obtained from tensile tests. The simulation results show that the outcomes align with those from the tests, and the identified errors can be kept under control.

## 1. Introduction

Additive manufacturing has experienced continuous, rapid, and diversified development in recent decades. This evolution has been made possible by major advancements in technique and technology during this period, which have enabled the design of new types of equipment, namely 3D printers. The wide variety of filaments and printer sizes has led to an increase in the number of users for additive manufacturing as well as the products obtained through this process. The development of the properties of 3D-printed structures, depending on the main printing parameters, is important for designers and those involved in finite element analysis.

Technological processes have had, and continue to have, a continuous evolution, as well as a positive impact on the development of human activities. The rapid progress of industry in the last century and the beginning of the current one generate challenges for designers regarding the design, processing, and optimization of various structures (parts). The mentioned optimization primarily refers to the selection of the material from which the structure will be processed, and secondly, to the choice of the most suitable technology for achieving the geometric shape defined by the designer. The improvement of the characteristics of manufactured structures (reducing material consumption while increasing performance) through various technologies would not be possible without the development of theoretical concepts for correctly and concretely addressing the issues that arise during the product life cycle.

The theoretical concepts (relations or hypotheses) are verified through laboratory tests, which sometimes confirm the theory, while other times they allow for its correction or modification. The differences between the theoretical approach and the test results can be evaluated, and depending on the obtained values, the theoretical concepts will be improved. Since theoretical models approximately (more or less) reflect reality, any differences are accepted in engineering practice. To obtain results with good accuracy, the model adopted by the designer must reflect, as closely as possible, the way the structure will behave in reality.

This study highlights the importance of correctly finding the mechanical and technological properties of 3D-printed materials from which the additively manufactured structures will be made. The major challenge is that the 3D printing process involves many parameters that need to be managed. Some of these parameters have a greater influence on the value of the mechanical properties, while others have a lesser influence. Furthermore, not all parameters have the same effect on the different mechanical properties. Correctly determining the mechanical properties is important because, based on these values, designers will size the structures they design. Moreover, without knowing the values of the mechanical properties, the analysis/modelling stage with finite element analysis (FEA/FEM) cannot be performed, especially since this tool is increasingly used by designers.

The configuration of 3D printing parameters can be performed within wide limits, sometimes without significantly affecting the value of the mechanical property. The customizing of the parameters requires performing tests to find the mechanical properties associated with those parameters, which involves a consumption of time and resources. A material commonly used due to its versatility in additive manufacturing is poly (lactic acid). The mechanical properties of poly (lactic acid), known as PLA, have been extensively studied, which is why there are works available that present information about the evolution of these properties.

In this study, researchers found that firstly tensile strength is affected by the infill percentage and, in a minor measure, by raster angle. The elastic modulus and fracture strain depend on the infill value and layer thickness. The best combination of the parameters is 80% for infill density and a 0.3 mm layer height [1]. Another paper shows that infill density has a considerable effect on mechanical properties, more precisely ultimate tensile strength and modulus of elasticity increase with infill density [2]. The type (manufacturer) of 3D printer has a small influence on the mechanical properties of 3D-printed PLA [3]. In another paper, the researchers showed that it is possible to optimize the printing parameters to obtain the desired mechanical properties [4]. For 80% infill density, the specimens with zigzag infill patterns have the highest tensile strength between other two different infill patterns (grid and concentric) [5]. If the printing parameters are the same, except the density and infill pattern, the highest values for Young’s modulus, yield strength, and ultimate tensile strength are for the concentric infill pattern. In second place is the grid pattern [6]. By increasing the infill density between 60 and 90%, the tensile strength also increases. Between three different infill patterns (gyroid, triangle, and grid), the best value for tensile strength is for the grid pattern [7]. Moreover, the compressive strength has the same evolution with ultimate tensile strength, which means that this increases with increasing infill density [8]. A study showed that the mechanical properties of PLA 3D-printed (ultimate tensile strength, Young’s modulus, and yield strength) had behaviour when the process parameters were changed. These conclusions were obtained by using ANOVA and Taguchi methods [9]. Also, the study showed that the tensile strength and elastic modulus decreased when the layer thickness increased [10]. In the case of 3D-printed PLA, for a printing temperature of 190 °C, layer height of 0.14 mm, and printing speed of 45 mm/s, the value of Poisson’s ratio increased as the filling density decreased [11]. This increase is almost linear, which allows the determination of the equation that establishes the link between the filling density and the value of the Poisson ratio. For PLA reinforced with carbon fibres (15% by mass), the Poisson’s ratio values were 0.400 and 0.163 [12]. For a printing temperature of 215 °C, printing layer height of 0.1 mm, printing speed of 45 mm/s, and printing angle of 45°, the Poisson’s ratio depended on the thickness of the test specimen [13]. More precisely, Poisson’s ratio is between 0.275 for the thickness of the specimen of 1 mm and 0.378 for the thickness of the specimen of 10 mm. For the printing temperature of 215 °C, the modulus of elasticity depends on both the printing angle and the height of the printed layer [14]. There are studies that show that the ultimate tensile strength is influenced by the printing temperature [15]. The influence is important when the printing temperature is between 190 °C and 200 °C. For temperatures between 200 °C and 230 °C, the value of the UTS oscillates a little. At 215 °C, the yield strength value is slightly influenced by the thickness of the specimen [13]. Also, compared to the maximum stress value, the yield strength value is, on average, 5% lower [12,16]. The studies showed that the elongation depends on the height of the printed layer, decreasing with the increase in the layer height [14]. Between 190 °C and 220 °C, the elongation changes by a maximum of 10% [15]. Furthermore, the research shows that the elongation depends on both the thickness of the specimen and the filling density [13,17]. Elongation increases with the increase in the thickness of the specimen but also with the increase in the filling density.

The purpose of this study is to determine and present the evolution of mechanical properties based on the printing temperature, for 3D-printed poly (lactic acid). Additionally, the influence of two other parameters was studied, specifically the infill density and printing speed. The mechanical properties are essential for conducting various studies, particularly in the case of numerical simulations (FEA/FEM) of additively manufactured structures, which cannot be performed without knowing the values of these properties. Moreover, the orientation of the gaps with one of their diagonals aligned with the longitudinal axis of the specimen expands the volume of available data regarding the 3D printing structures of poly (lactic acid). The complex geometry of the specimens with 75% and 50% infill density allows testing of the simulation process for these, namely, if the process can be applied with acceptable results. The novelty lies in the fact that the evolution of mechanical properties is based on a large number of tests conducted on additively manufactured PLA specimens. Researchers have conducted similar studies on mechanical properties, but they were based on the application of the Taguchi method or analysis of variance. Furthermore, the evolution of certain mechanical properties is presented as function of two printing parameters. This type of presentation helps with understanding how the respective property evolves with printing parameters. Additionally, based on the results of the mechanical tests and statistical analysis (multiple linear regression), the graphs are accompanied by equations that allow the determination of property values as a function of printing parameters. Another purpose of this study is to compare the results obtained through finite element analysis with those derived from mechanical tests conducted on 3D-printed PLA specimens.

## 2. Theoretical Considerations

**Figure 1:**
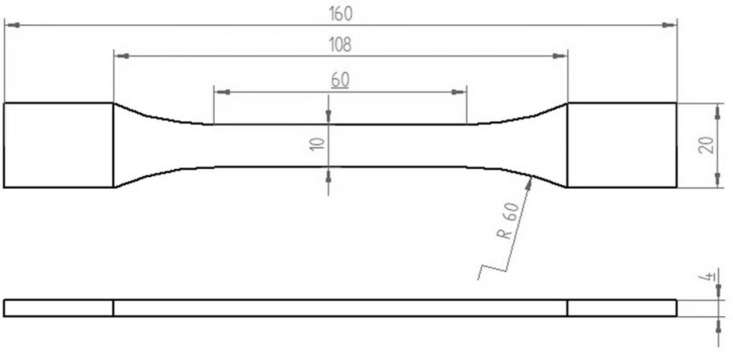
Geometry and dimensions of 3D printed PLA specimens.

The mechanical properties of materials are characteristics that differentiate how they respond to various loads acting upon them. The most used test for determining the main mechanical properties is the tensile test. This test experimentally establishes the physical relationship between stresses and strains under tensile loading. The tensile testing procedure is governed by established standards. In this study, tensile tests were conducted on specimens manufactured in accordance with the standard ISO 527-2 second edition 2012. The geometric shape and dimensions of the additively manufactured specimen are illustrated in Figure 1.

**Figure 2:**
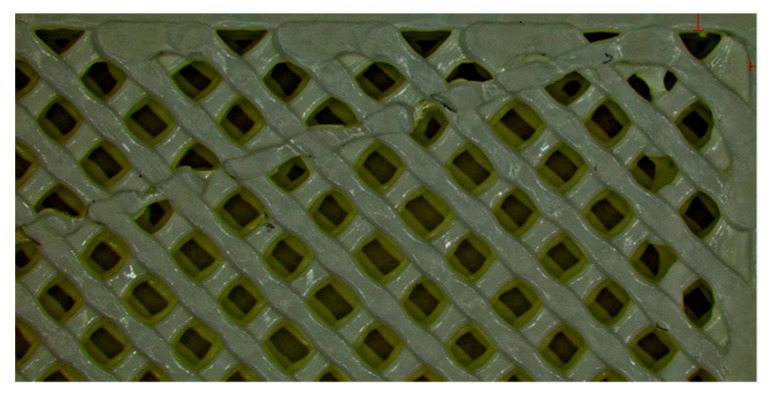
Shape and arrangement of gaps for specimens with infill densities less than 100%.

The specimens were printed with densities of 100%, 75%, and 50%. For specimens with densities of 75% or 50%, the gaps (voids) are square-shaped and oriented with a 45° rotation relative to the longitudinal axis of the specimen (see Figure 2). Specifically, one diagonal of the square is parallel to the longitudinal axis of the specimen, while the other diagonal is perpendicular to it.

**Figure 3:**
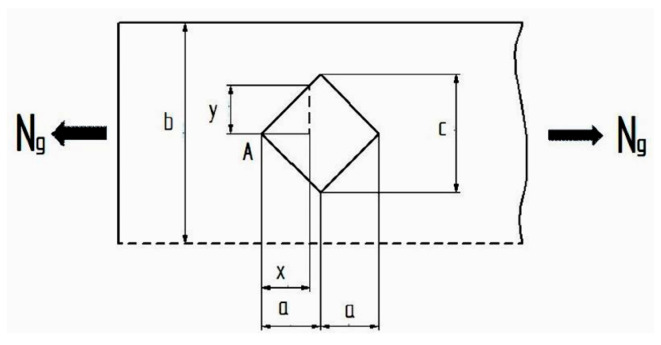
Isolated part of the specimen.

The tensile behaviour of specimens with a filling density less than 100% is complex due to the presence of gaps and the fact that the printing angle is 45° relative to the longitudinal axis of the specimen. In Figure 3, one of the many gaps in the specimen is isolated, and its dimensions and the way forces act upon it are illustrated.

The stress in the region of the specimen that contains the gap is variable, reaching its maximum value at the midpoint of the gap. Specifically, the stress begins to increase at the start of the gap (point A). Once the midpoint of the gap is passed, the stress begins to decrease again. The relationship for calculating the stress is:

σ = N_g_/S = N_g_/((b − 2y)h) = N_g_a/((ba − cx)h) (1)

where σ is the tensile strength acting on the respective region, N_g_ represents the force applied to the stressed region (gap region), a is the distance from point A to the diagonal of the square, b represents the width of the stressed region, c is the diagonal of the gap (square), h is the height of the 3D-printed specimen (4 mm), x represents the distance from corner A, and S is the surface of the transversal stressed region.

The behaviour described in Figure 3 and expressed by Equation (1) is repeated for each gap in the specimen, both along its length and width. During the tensile test, due to the applied force, the specimen elongates until it fractures. In terms of elongation (elongation at break), this is calculated using Equation (2) for regions without gaps and Equation (3) for regions containing gaps:

Δl = (N_g_a)/(ES) = (N_g_a)/(Ebh) (2)

(3) $$\int_{0}^{a}\left(N_{g}\mathrm{dx})/(\mathrm{ES})=((N_{g}a)/(\mathrm{Eh}))\int_{0}^{a}\mathrm{dx}/(\mathrm{ba}-\mathrm{cx})=((N_{g}a)/(\mathrm{Ehc}))\ln(b/(b-c)\right)$$

where E is the modulus of elasticity. The relationships presented are valid only for gaps that have a square shape and no rounded corners. It should be noted that the gaps in the 3D-printed specimens are not perfectly square (they exhibit small deviations from the intended shape). Additionally, the dimensions of the squares vary from one gap to another. In practice, the shape and dimensions are not ideal (see Figure 2). Furthermore, if the gap has a different geometric shape and possibly rounded corners, relationships (2) and (3) are no longer valid. However, it is possible to determine new relationships for the new geometry. Regardless of the infill density (50% or 75%), the relationships are still valid, as only the dimensions of the gaps and the frequency of their repetition along the length and width of the specimen change.

## 3. Experimental Setup

For the 3D printing of the specimens tested under tensile stress, poly (lactic acid) filament manufactured by Suntem3D was used, with the following mechanical properties:

- Filament diameter 2.85 mm;
- Tensile strength 110 MPa (ASTM D882);
- Impact resistance 7.5 kJ/m^2^ (ISO 180/A);
- Modulus of elasticity 3310 MPa (ASTM D882);
- Bending modulus of elasticity 2392.5 MPa.

More technical characteristics regarding the filament used for the fabrication of the specimens can be found in the technical data sheet. The specimens made from the mentioned filament were printed using a CreatBot DX Plus printer with dual print heads. The printer specifications are as follows:

- Printing dimensions 300 × 250 × 520 mm;
- Filament diameter 2.8–3 mm;
- Layer resolution 0.2 mm;
- Print nozzle size 0.2–0.8 mm;
- Printing resolution 0.6 mm;
- Printing volume 39 l.

For all the printed specimens, the printing parameters were as follows:

- Layer height 0.2 mm;
- Bed temperature 61 °C;
- Printing angle 45°;
- Infill overlap 10%;
- Infill flow 110%.

The specimen codes, based on the 3D printing parameters, are presented in Table 1. The 3D printing parameters that were variable during the processing of the specimen were:

- Printing temperature 200 °C, 210 °C, 220 °C, or 230 °C;
- Infill density 100%, 75%, or 50%;
- Printing speed 40, 60, or 80 mm/s.

The solid infill at the top and bottom 1 mm were used for an infill density of 75 and 50%. The additively manufactured specimens were the same type used for tensile testing and had geometry and dimensions in accordance with the standard ISO 527-2 second edition 2012 (specimen type (ISO 527-2/1B/5).

## 4. Results and Discussions

After the additive manufacturing of the poly (lactic acid) specimens, they were subjected to tensile tests.

The tensile tests were performed on a WDW-150 S universal testing machine (applied test force varied between 0.1 and 150 kN) and testing conditions were: tighten speed 5 mm/min and stress speed 10 MPa/s. The results of the tensile tests conducted on the 3D-printed specimens are presented in Table 2. For each specimen code, 5 specimens were 3D-printed, and the results obtained fell within the confidence interval set at 95%.

**Figure 4:**
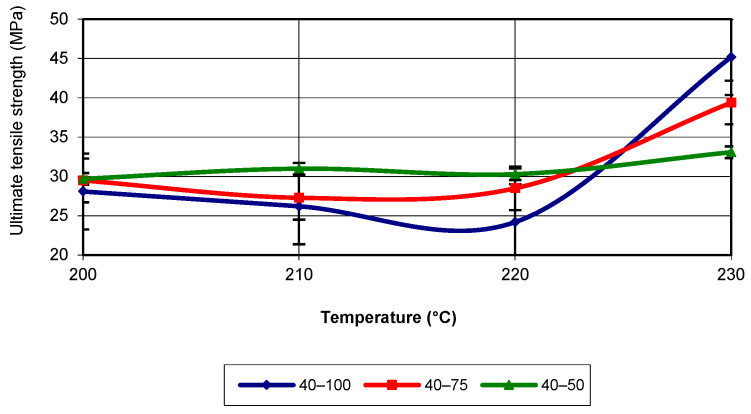
Evolution of ultimate tensile strength as a function of printing temperature (v = 40 mm/s).

The first mechanical property studied for 3D-printed poly (lactic acid) was the ultimate tensile strength (UTS) or tensile strength (TS). Without this main property, as well as others, finite element analysis cannot be performed. The evolution of this property was analysed in terms of the influence that the main parameters of additive manufacturing have on it. The main parameters considered were printing speed, infill density, and temperature. Figure 4 shows the evolution of tensile strength as a function of printing temperature for a printing speed of 40 mm/s.

Analysing the figure, it can be observed that the maximum value of tensile strength is obtained at a printing temperature of 230 °C and an infill density of 100%. In general, for printing temperatures of 200 °C, 210 °C, and 220 °C, the tensile strength is higher for an infill density of 50%. In the same temperature range, the lowest values are recorded for an infill density of 100%. Between 220 °C and 230 °C, the behaviour of the property is reversed. The figure shows a slight fluctuation in the property values up to 220 °C, after which a sharp increase is observed. The equation that characterizes the evolution of tensile strength is neither linear nor parabolic but corresponds to a third-degree polynomial equation. Regardless of the infill density, this type of evolution best fits the values obtained in the tensile tests (R-squared value of 1).

**Figure 5:**
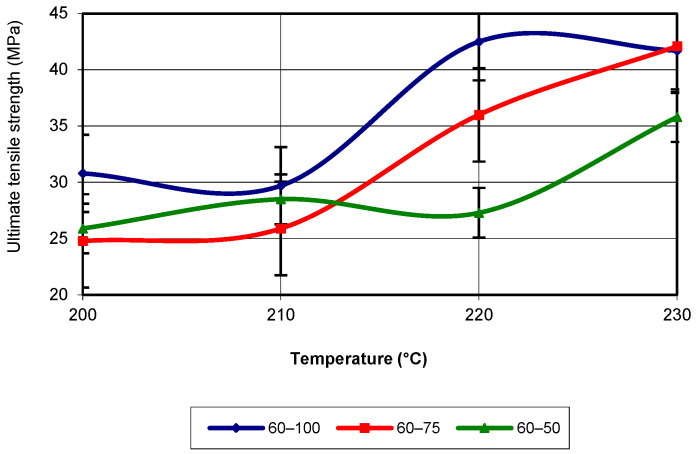
Evolution of ultimate tensile strength as a function of printing temperature (v = 60 mm/s).

For a printing speed of 60 mm/s, the evolution of tensile strength changes, meaning that the best values of the analysed property across the entire temperature range are achieved for an infill density of 100% (see Figure 5). Additionally, for an infill density of 75%, the values are higher than those for 50% in the temperature range of 200 °C to 230 °C. The evolution of the analysed property for all infill densities still corresponds to a third-degree polynomial equation, with an R-squared value of 1.

**Figure 6:**
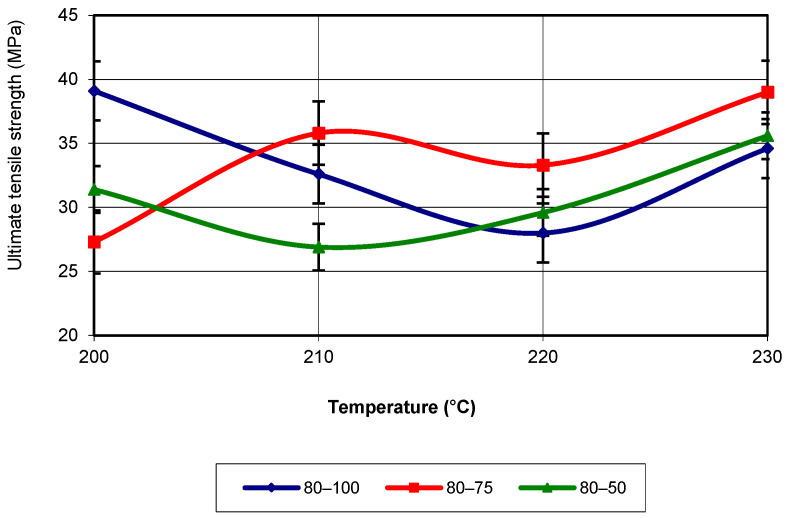
Evolution of ultimate tensile strength as a function of printing temperature (v = 80 mm/s).

For a printing speed of 80 mm/s, the tensile strength reaches its highest value at a printing temperature of 200 °C. For the other temperatures, the highest tensile strength values are recorded for an infill density of 75% (see Figure 6). For infill densities of 100% and 50%, the tensile strength values are quite similar.

If the evolution of the studied property is analysed, it again corresponds to a third-degree polynomial equation with an R-squared value of 1. For ultimate tensile strength, at print speeds of 40 and 60 mm/s, the best results are obtained at temperatures of 220 °C and 230 °C. Due to the higher temperatures, the filament adheres better to the existing layers. When the speed is 80 mm/s, the filament does not have enough time to adhere to the already deposited layers, which is why the UTS values are not as high as for the other speeds.

**Figure 7:**
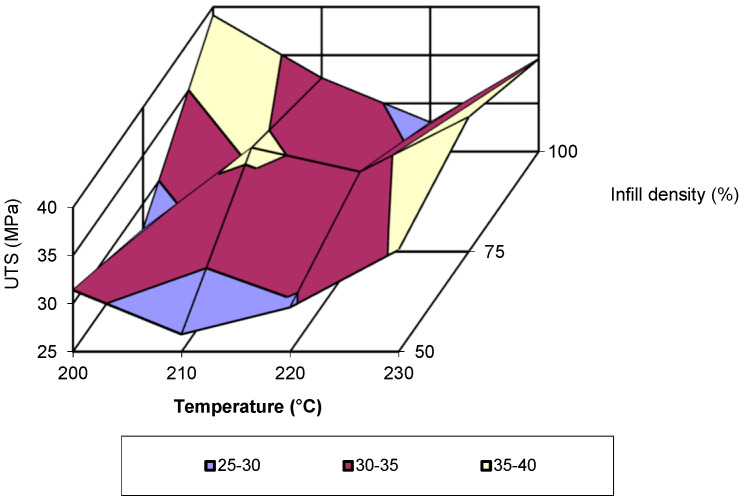
Evolution of ultimate tensile strength as a function of printing temperature and infill density (v = 80 mm/s).

In Figure 7, the evolution of tensile strength as a function of printing temperature and infill density is presented. This representation of the test results is visually effective and allows for an easy understanding of the evolution of the analysed property. However, the figure has the disadvantage of not including all the parameters involved in 3D printing. Specifically, it presents values only for a printing speed of 80 mm/s. Analysis of the figure shows that for the mentioned speed, the highest tensile strength value is recorded at a printing temperature of 200 °C and an infill density of 100%. The lowest values are recorded for a temperature of 210 °C and an infill density of 50%. The third-degree polynomial equations that describe the evolution of tensile strength as a function of temperature and are valid for all printing speeds align with the results obtained in previous studies on the additive manufacturing of poly (lactic acid) [16,18,19,20,21,22], if they are processed accordingly. Analysing the data from these studies, it can be observed that the evolution of tensile strength follows about the same third-degree polynomial equation.

**Figure 8:**
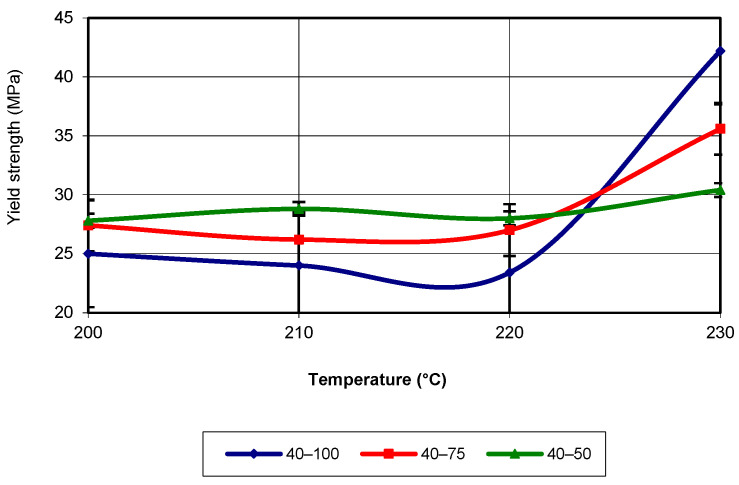
Evolution of yield strength as a function of printing temperature (v = 40 mm/s).

Another property required for the simulation process is the yield strength. When this property value is reached, the material of the specimen, and essentially of any structure, begins to yield. In other words, the specimen does not break, but it starts to degrade, and any increase in the applied load beyond the one that initiated this degradation will lead to the potential failure of the specimen. Figure 8 presents the evolution of yield strength as a function of temperature for a printing speed of 40 mm/s.

**Figure 9:**
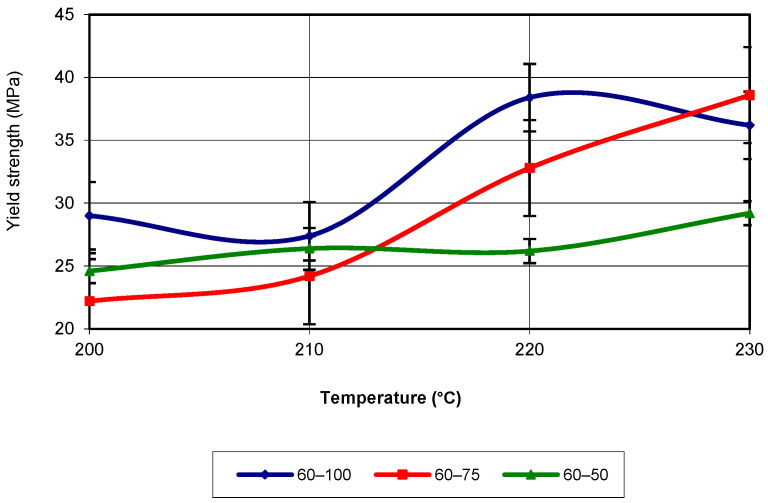
Evolution of yield strength as a function of printing temperature (v = 60 mm/s).

The analysis of the figure shows that for temperatures below 220 °C, the highest values of yield strength were recorded for specimens with an infill density of 50%, followed by those with an infill density of 75%. At a printing temperature of 230 °C, specimens with an infill density of 100% recorded the highest values. The equations that characterize the curves in the figure are third-degree polynomial equations with an R-squared value of 1. When the printing speed is 60 mm/s (see Figure 9), the yield strength values are highest for an infill density of 100% up to temperatures close to 230 °C. Near the mentioned temperature, for an infill density of 75%, the highest yield strength value is recorded. In terms of the evolution trend, this corresponds to a third-degree polynomial equation, with an R-squared value of 1.

**Figure 10:**
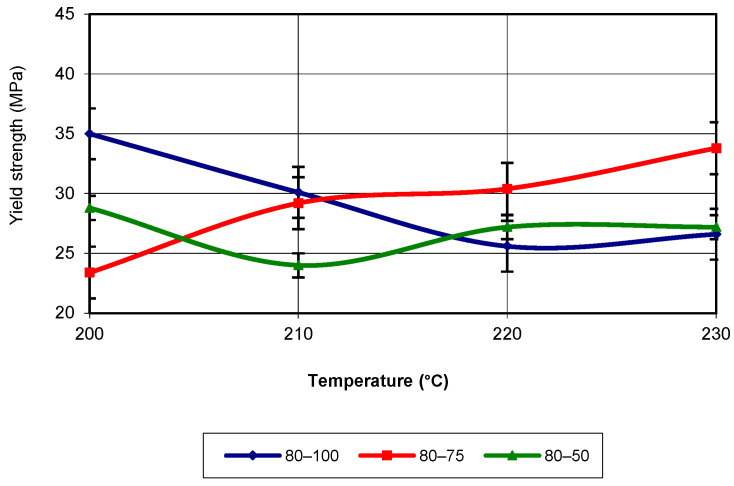
Evolution of yield strength as a function of printing temperature (v = 80 mm/s).

**Figure 11:**
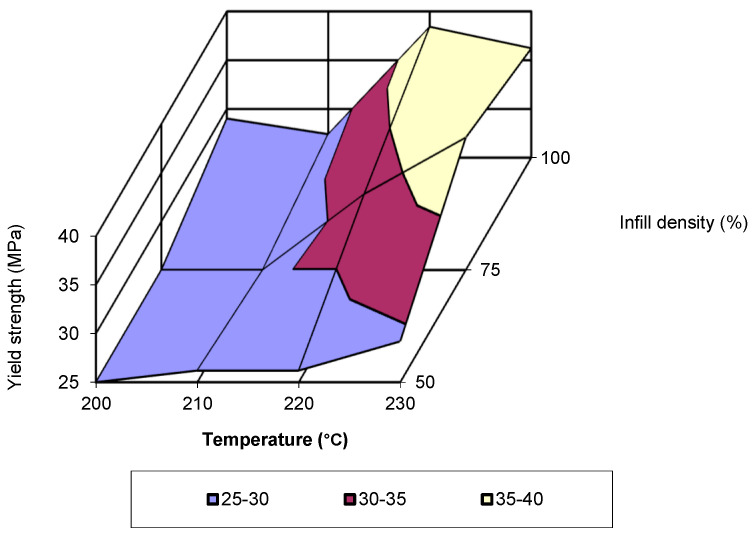
Yield strength as a function of printing temperature and infill density (v = 60 mm/s).

For a printing speed of 80 mm/s, the evolution of yield strength as a function of printing temperature is presented in Figure 10. Between 200 °C and 210 °C, specimens printed with an infill density of 100% recorded the raised values for yield strength, while for the other temperatures, the raised values were recorded for an infill density of 75%. In terms of the evolution trend, this corresponds to a third-degree polynomial equation, with an R-squared value of 1. Figure 11 presents the evolution of yield strength as a function of the two printing parameters (temperature and infill density) for a printing speed of 60 mm/s.

**Figure 12:**
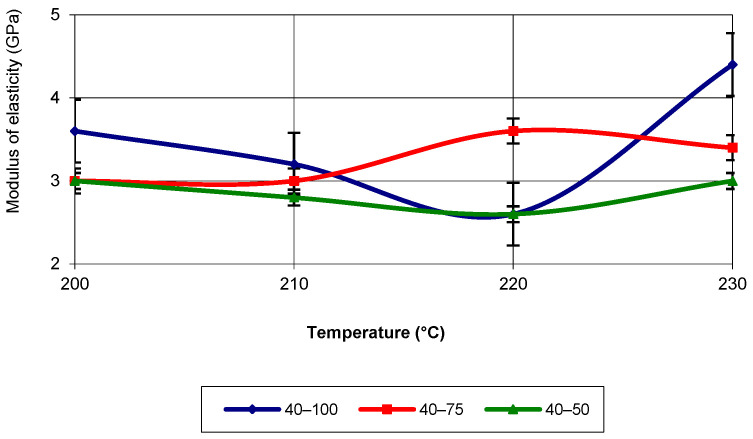
Elastic modulus as a function of printing temperature (v = 40 mm/s).

The analysis of the figure shows that the lowest values are recorded for temperatures of 200 °C and 210 °C, with infill densities of 50% and 75%. The highest value is recorded for a temperature of 220 °C and an infill density of 100%. By analysing the data from the studies conducted over time [1,16,21,23,24,25] and the adequate processing of the data provided by them, it is observed that the evolution of yield strength follows a third-degree polynomial equation. The elastic modulus, also known as modulus of elasticity or Young’s modulus, is another property necessary for the simulation process. For a printing speed of 40 mm/s, the highest values were recorded for an infill density of 100% across almost the entire temperature range (see Figure 12).

**Figure 13:**
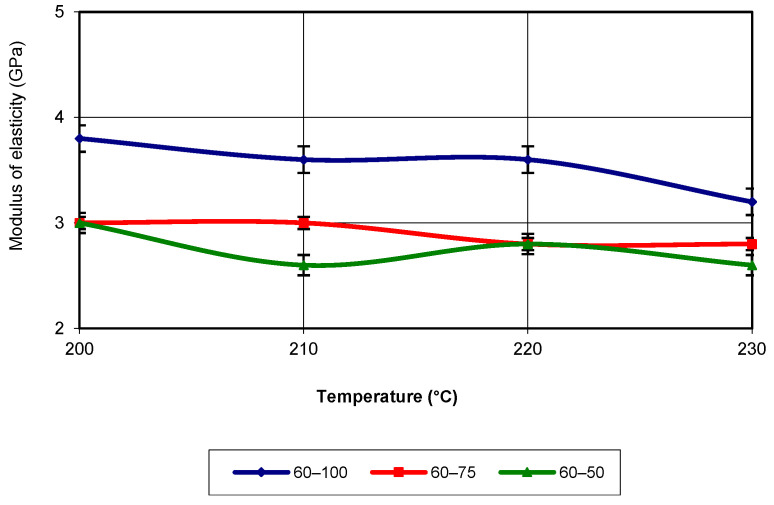
Elastic modulus as a function of printing temperature (v = 60 mm/s).

In the temperature range of 210 °C and 220 °C, the highest values were recorded for the specimens with an infill density of 75%. At a printing speed of 40 mm/s, the elastic modulus had the lowest values for specimens printed with a 50% infill density. In terms of evolution, the elastic modulus follows a third-degree polynomial equation with an R-squared value of 1. At a printing speed of 60 mm/s, the values of the elastic modulus are the highest for specimens with 100% infill density (see Figure 13). This behaviour is present across the entire temperature range, with values significantly higher than those recorded for infill densities of 75% and 50%.

**Figure 14:**
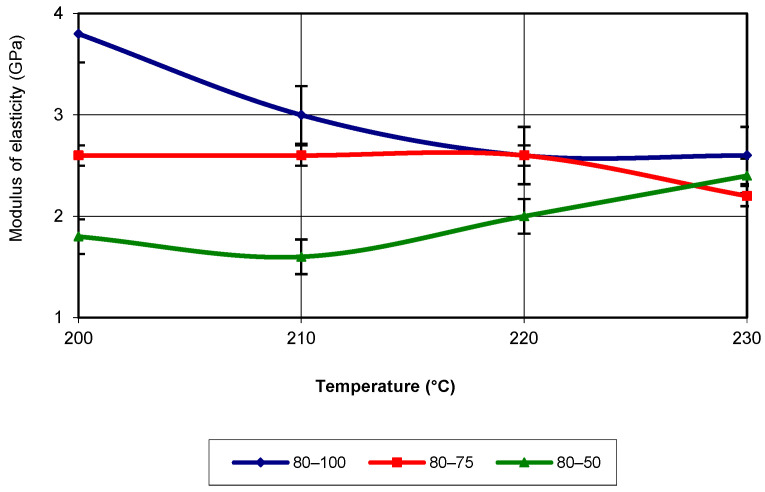
Elastic modulus as a function of printing temperature (v = 80 mm/s).

The equation that characterizes the evolution of the property corresponds to a third-degree polynomial with an R-squared value of 1. At a printing speed of 80 mm/s, for 100% infill density, the elastic modulus registers the highest values across the entire temperature range of 3D printing (see Figure 14).

The lowest values of this property are recorded for specimens printed with 50% infill density. As with the previously analysed printing speeds, the evolution also follows a third-degree polynomial equation with an R-squared value of 1.

**Figure 15:**
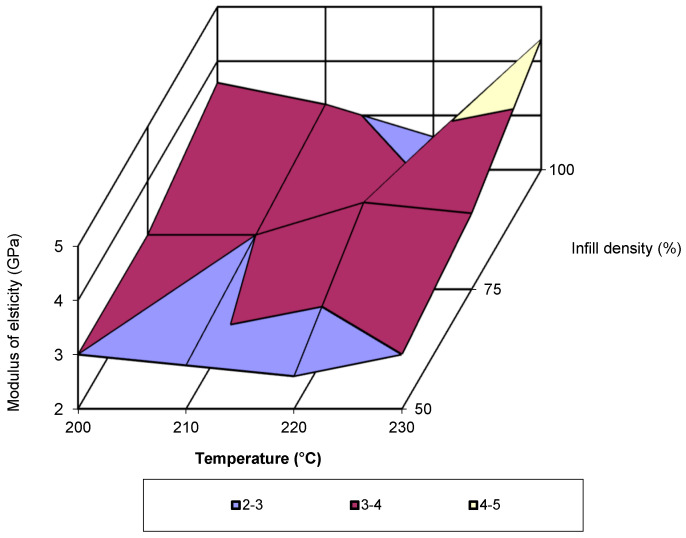
Elastic modulus as a function of temperature and infill density (v = 40 mm/s).

In Figure 15, the evolution of the elastic modulus is presented for a print speed of 40 mm/s as function of the two printing parameters (temperature and infill density). Analysis of the figure shows that the lowest values are recorded at a temperature of 220 °C and infill density of 50% and 100%. The highest value is recorded at temperature of 230 °C with an infill density of 100%. By analysing the data from previous studies [11,13,15,26,27] and the adequate processing of the data provided by them, it is observed that the evolution of the elastic modulus follows a third-degree polynomial equation.

**Figure 16:**
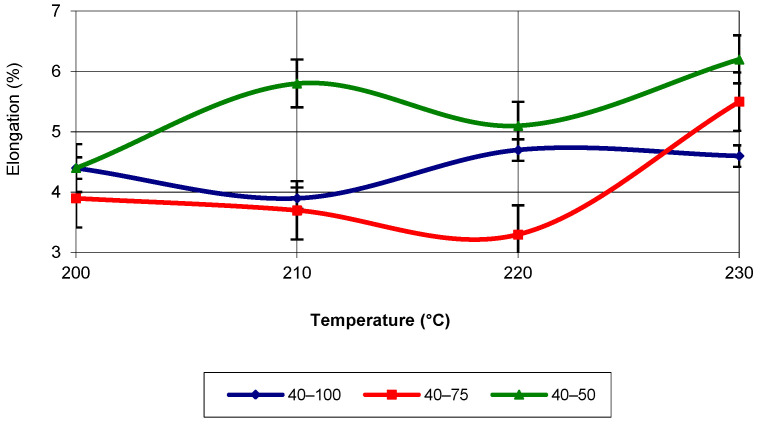
Elongation as a function of printing temperature (v = 40 mm/s).

Another property necessary for the simulation process is the elongation at break (elongation). Figure 16 presents its evolution for a print speed of 40 mm/s. The highest values for all temperatures are recorded for an infill density of 50%, with a maximum value at a temperature of 230 °C. The lowest values are recorded for an infill density of 75% for all temperatures, except for 230 °C.

**Figure 17:**
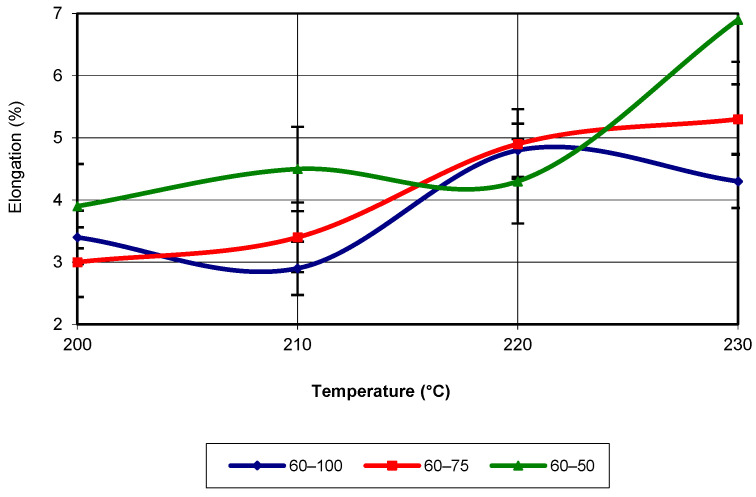
Elongation as a function of printing temperature (v = 60 mm/s).

The equation that characterizes the evolution of elongation is not linear or parabolic but corresponds to a third-degree polynomial equation. Regardless of the infill density, this type of evolution best matches the values obtained from the tensile tests (with an R-squared value of 1). Figure 17 shows the evolution of elongation as a function of temperature, for a print speed of 60 mm/s.

**Figure 18:**
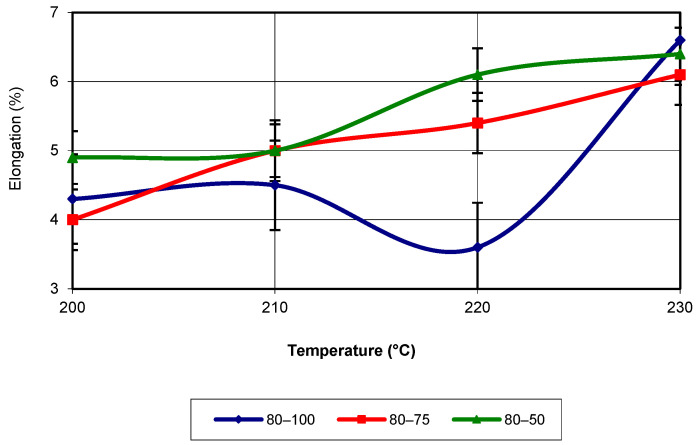
Elongation as a function of printing temperature (v = 80 mm/s).

**Figure 19:**
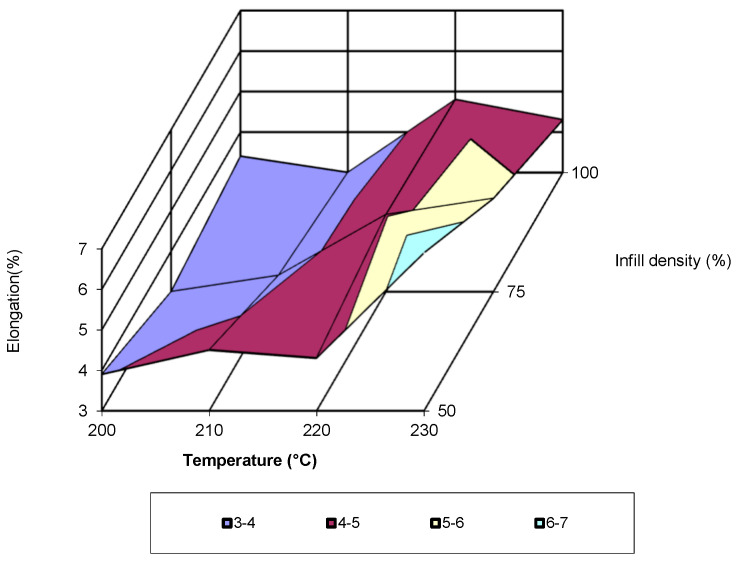
Elongation as a function of temperature and infill density (v = 60 mm/s).

Analysis of the figure shows that almost across the entire temperature range, the highest values of the property are associated with an infill density of 50%. Around the temperature of 220 °C, the specimens printed with a density of 75% exhibit the highest values. The maximum values occur at a temperature of 230 °C. The equation that characterizes the evolution of this property is a third-degree polynomial, with an R-squared value of 1. For a printing speed of 80 mm/s, the evolution of elongation proceeds similarly to the previously analysed speeds. Regardless of the additive manufacturing temperature, the highest values are recorded for the specimens printed with a 50% infill density (see Figure 18). Additionally, the maximum values are obtained at a temperature of 230 °C, regardless of the infill density. The equation that characterizes the evolution of elongation is of the third-degree polynomial type, with an R-squared value of 1. In Figure 19, the evolution of the elongation as a function of the two printing parameters (temperature and infill density) for a printing speed of 60 mm/s is shown.

The elongation for the specimens with infill density of 50% had the best behaviour for almost temperatures and printing speeds. The explanation is that the presence of gaps leads to an increase in this property, namely the structure becomes more plastic. Also, for the infill density of 75%, for the speed of 60 and 80 mm/s, the elongation has a favourable evolution compared to the structure with an infill density of 100%.

Analysis of the figure indicates that the lowest values are recorded at a printing temperature of 210 °C and an infill density of 100%. A value close to the minimum is also recorded at a temperature of 200 °C and an infill density of 75%. The highest value is recorded for a temperature of 230 °C and an infill density of 50%. By analysing data from earlier studies [15,17,28,29] and the adequate processing of the data provided by them, it is observed that the evolution of the modulus of elasticity follows a third-degree polynomial equation.

## 5. Results of Statistical Processing and Numerical Simulation

In the previous section, the results of the tests conducted were presented. These results show how the studied properties evolve depending on the parameters of additive manufacturing. While in the case of property evolution based on a single printing parameter, the equation that characterizes the evolution is a third-degree polynomial. In the case of evolution based on two parameters, finding a mathematical solution is challenging. The main purpose of this study is as the established property values to be used in the finite element analysis of the behaviour under various loads of the additively processed structures. For this approach, presenting the analysed properties based on one or two printing parameters is not the ideal solution for numerical simulation. A possible solution to address this issue is the use of multiple linear regression. Although this is not the ideal option as it can introduce errors (sometimes significant), it allows the calculation of the property values based on the parameters with which the specimens used in the current study were printed.

In the case of multiple regression, there is one dependent variable and several independent variables. The purpose of multiple linear regression is to establish the relationship between a dependent (outcome) variable and a set of independent (factorial) variables. Consider a set of *n* random variables *X*_1_, *X*_2_, …, *X*_n_ which are assumed to be independent and that from the probabilistic point of view represent the variables that influence a process. These variables act independently of each other. Another random variable, denoted by *Y*, represents the dependent variable, whose values will be estimated based on multiple linear regression. The relationship for multiple linear regression is of the form:

*Y* = a + b_1_X_1_ + b_2_X_2_ + … + b*_n_*X*_n_*, (4)

where a is intercept and b_1_, b_2_, …, b_n_ are regression coefficients. The variables involved in regression must meet the following conditions:

- The relationship between the dependent variable and the independent variable must be linear;
- The effects of each variable are, in principle, independent of the others.

Multiple linear regression allows, in certain cases, the hierarchical ranking of the influence of independent variables on the dependent one. The analysis is performed to retain only the variables that have a significant impact on the dependent variable, discarding those that are insignificant. For this ranking, the significance level (*p*-value) is studied, which should be below 5%. The statistical term *p*-value indicates whether there is a real relationship between the independent and dependent variable, or in other words, whether the independent variable has a statistically significant relationship with the dependent variable, even after accounting for the other variables in the model. The smaller the *p*-value (threshold 0.05), the more likely it is that a real relationship exists between the independent and the dependent variable. In this study on the additive manufacturing of poly (lactic acid), the parameters involved in printing (speed, infill density, and temperature) are considered independent variables.

The first property for which multiple linear regression is determined is ultimate tensile strength. Based on the data in Table 2, by studying the correlation coefficient, it is observed that there is a reasonable correlation (0.6 value of the coefficient) between tensile strength and printing temperature. Between tensile strength and infill density, there is a weak correlation, as the correlation coefficient is 0.2. For ultimate tensile strength (UTS), the multiple linear regression relationship is:

UTS = −36.2675 + 0.04333 v + 0.0625 d + 0.2842t(5)

where v, d, and t are printing speed, infill density, and temperature. The indicators generated along with the linear regression show that the model is globally significant, as it has a Significance-F value of 0.000855. The statistical term Significance-F provides an overall assessment and, at the same time, is a critical indicator of the validity of the multiple linear regression model. It shows whether the independent variables have a significant impact on the dependent variable. Furthermore, the *p*-value indicator is 0.02222 for the intercept and 0.000199 for temperature. These values indicate that tensile strength is primarily influenced by printing temperature, followed by infill density, and finally by printing speed (with *p*-values of 0.1015 and 0.3567, respectively). Also, the adjusted R^2^ is 0.343957 and standard error is 4.5404143. If the tensile strength values are calculated with relationship (5), the errors average between the calculated values and those obtained in the tests is 2%.

In the case of yield strength, based on the data in Table 2, the correlation coefficient is 0.5 for temperature, indicating a reasonable correlation. The correlation coefficient for infill density is 0.2, indicating a weak correlation. For determining the yield strength (YS), the multiple linear regression relationship is:

YS = −18.6964 − 0.0075v + 0.059167d + 0.203t(6)

The indicators generated along with the multiple linear regression show that the model is globally significant, as it has a Significance-F value of 0.008661. Furthermore, the *p*-value indicator is 0.185327 for the intercept and 0.002526 for temperature. These values indicate that yield strength is primarily influenced by printing temperature, followed by infill density, and finally by printing speed (with *p*-values of 0.09075 and 0.8607, respectively). The adjusted R^2^ is 0.236053814 and the standard error is 4.154937345. If the yield strength values are calculated with relationship (6), the errors average between the calculated values and those obtained in the tests is 2%.

From the perspective of the modulus of elasticity, the correlation coefficient is −0.1 for temperature, meaning that a weak correlation range is not achieved. In contrast, for infill density and speed, the correlation coefficients are 0.6 and −0.5, indicating a reasonable correlation. To determine the value of the modulus of elasticity (ME), the multiple linear regression relationship is:

ME = 3.5 − 0.0175v + 0.01633d − 0.00356t(7)

The indicators generated along with the linear regression show that the model is globally significant, as it has a Significance-F value of 1.48 × 10^−6^. Furthermore, the *p*-value indicator has a value of 0.009337 for the intercept and 9.46 × 10^−6^ for infill density. These values indicate that the modulus of elasticity is primarily influenced by infill density, followed by speed, and lastly by printing temperature (*p*-value 8.34 × 10^−5^ and 0.535385, respectively). Also, the adjusted R^2^ is 0.5641189 and the standard error is 0.380665536. If the elastic modulus values are calculated with relationship (7) the errors average between the calculated values and those obtained in the tests is 2%.

The last property of additive manufactured poly (lactic acid) for which the correlation coefficient is determined is elongation at break. The correlation coefficient is 0.7 for temperature, indicating a high correlation. For infill density, the correlation coefficient is −0.4, which suggests a reasonable correlation. The correlation coefficient for printing speed is −0.1, signifying a very weak correlation. To determine the elongation at break (E), the multiple linear regression relationship is:

E = −6.49056 − 0.00813v − 0.0205d + 0.0601t.(8)

The indicators generated along with the multiple linear regression show that the model is globally significant, as it has a Significance-F value of 5.81 × 10^−7^. Furthermore, the *p*-value indicator is 0.005792 for the intercept and 7.96 × 10^−7^ for temperature. These values indicate that elongation at break is primarily influenced by printing temperature, followed by infill density, and lastly by printing speed (*p*-value 0.000608 and 0.236764, respectively). The adjusted R^2^ is 0.589265718 and the standard error is 0.660254318. If the elongation values are calculated with relationship (8), the errors average between the calculated values and those obtained in the tests is 2%.

**Figure 20:**
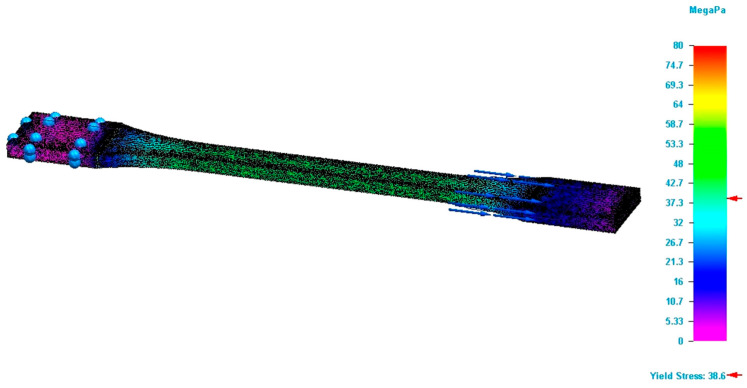
Result of the simulation process for the T673 specimen (Von Mises stress).

The values of the properties of 3D-printed poly (lactic acid) are useful in the design of structures to be additively manufactured and in the process of simulating the behaviour of these structures under various loads. In the case of the boundary conditions, the simulation was carried out under conditions similar to those of a tensile test, since only the results from those tests were used. Specifically, the process replicates the tensile test, meaning that one end of the specimen is considered fixed while the other end moves under the action of the tensile force. No additional displacement conditions were imposed. Regarding temperature conditions, it was the same as that at which the properties of the 3D-printed PLA were established (ambient temperature). For the loading conditions, it was assumed that the tensile force is applied along the longitudinal axis of the specimen. Furthermore, the load is applied on the wide section of the specimen. Loads in other directions were not considered. The mesh size was used as the convergence criterion. Prior to the simulation, tests were conducted with different mesh sizes, and the following mesh dimensions were established as providing sufficiently accurate solutions: 1.8 mm for specimens with 100% infill density, and 0.26 mm and 0.37 mm for those with 75% and 50% infill density, respectively. For all simulations, the value of Poisson’s ratio was the same for the longitudinal and transversal direction. Through the simulation process, designers can improve the performance of these structures under different loads. The property values obtained from tensile tests on 3D-printed poly (lactic acid) specimens serve as input data for the simulation process. The applicability of simulation to 3D-printed poly (lactic acid) is verified by the results provided by the finite element analysis process. Specifically, for each 3D-printed specimen, the mentioned verification is performed, considering that the specimen is subjected to the maximum force at which it broke during the test. Additionally, the input data include the modulus of elasticity, yield strength, and elongation at break taken from Table 2, but also the dimension of gaps and their arrangement in case of the infill density of 75% and 50%. Other properties necessary for the simulation process (density of 3D-printed structures and Poisson’s ratio) were taken from the previously performed studies or from the published studies. Based on these values, the simulation process provides the Von Mises stress value developed in the specimen (see Figure 20). This value should be equal to or close to that obtained from the testing. Figure 20 shows the final stage of the simulation. Moreover, the study type is linear static with quadratic tetrahedral mesh type.

The results of the simulation process are presented in Table 3. The analysis of differences between the values obtained during the simulation process and those recorded during the tests reveals that the errors range from 3.8% to 19.3%. Specifically, the values determined through simulation are higher than those observed during the tensile tests. This discrepancy can be explained by the fact that 3D-printed specimens are not perfect (see Figure 2). These imperfections weaken the material, as highlighted during the tensile tests.

**Figure 21:**
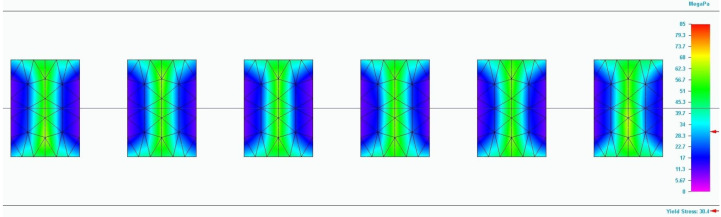
Result of the simulation process for the T453 specimen (longitudinal section through middle of the gaps).

The simulation results in the column ultimate tensile strength (UTS) in the gap show how the Von Mises stresses are distributed in the gap areas of the 3D-printed specimen with an infill density of 50% (see Figure 21). Analysis of the figure shows that the maximum stresses occur in the middle of the gap, as observed in Figure 21. This behaviour was anticipated by the relationship (1) and confirmed by the simulation process. The simulation demonstrates that lower stresses are present at the beginning of the gap, reaching their maximum value in the middle (61.0 MPa).

Although there are peak values along the diagonal of the gap, the average of all values in the gap area generated by the simulation process is lower than the stress at which the specimen failed. This behaviour was observed in all specimens with gaps, specifically those 3D-printed with an infill density of 75% and 50%.

## 6. Conclusions

This study refers to the 3D printing of the poly (lactic acid) using filament with a diameter of 2.85 mm. Moreover, the study takes in consideration the most common values used in PLA printing for the analysed parameters, which is why wide ranges resulted. The advantages of these are that potential users will find the values of the properties of additively manufactured structures for the chosen printing parameters, without having to perform the specific tests.

The properties evolution as a function of temperature was analysed exhaustively because this parameter had the greatest influence on the analysed properties. Also, this study showed that when discussing the evolution of the analysed properties (ultimate tensile strength, yield strength, modulus of elasticity and elongation at break) as a function of printing temperature, all of these can be characterized by third-degree polynomial equations. The positive aspect of expressing the relationship between the property and the parameter through a polynomial equation is the high accuracy of the calculated value for the analysed property. The calculated value is close to that obtained in the tests. The negative aspect of polynomial equations is that for each property, printing speed, and infill density, the corresponding equation must be determined. This approach is difficult (cumbersome) because the user of this information needs to be highly attentive when selecting the correct equation based on the values of the printing parameters. In the case of expressing property values as a function of two parameters (temperature and infill density), three such representations are needed for each property, but they do not provide the accuracy of polynomial equations.

The solution for integrating all 3D printing parameters into a single equation is to use statistical processing. Statistical processing shows that ultimate tensile strength, yield strength and elongation depend on temperature and infill density. Printing speed has a minimal influence on these properties. For the modulus of elasticity, statistical processing indicates that it is primarily influenced by infill density, printing speed, and, lastly, by printing temperature. Moreover, for the ultimate tensile strength, yield strength, and modulus of elasticity, the lowest error between the values calculated with relationships (5)–(7) and those of the tests was obtained for the specimen code T810. The highest error was obtained for the specimen with the code T412. In the case of elongation at break calculated with relationship (8), the lowest error was obtained for the specimen T410 and the highest for the specimen T472. Multiple linear regression equations can be easily integrated into the material property database used by the simulation software. Thus, after integrating the linear regression equations into the simulation program, based on the values of the additive manufacturing parameters, the program will provide the property values required for the simulation process. This approach helps designer’s work in obtaining the desired characteristics for the target benchmarks.

The manuscript shows that regardless the infill density values used in 3D printing, the errors recorded by the simulation process are in line with those of the tests but also considering the deviation of the specimens, from the ideal geometry. In the case of specimens with 100% infill density, the analysis of the simulation results shows that the determined ultimate tensile strength is up to 4.1% higher than that obtained in the tests. For specimens with 75% or 50% infill density, the simulation results are up to 19.3% and 19% higher, respectively, than those recorded in the tests. To ensure the operational safety of 3D-printed structures and avoidance of their failure during loading, the yield strength obtained in tests can be multiplied by a safety factor (reserve), which is sub unitary. This value could be 0.75 to account for all errors due to material properties or the simulation program. The use of such a coefficient is commonly met in the tensile strength calculations of structures made from alloys. In this way, the imperfections of the printed structures and any deviations in the printing parameters from the configured values due to unpredictable factors will be covered by this safety factor. Thus, the results of the simulation process will align with those obtained in the tests, increasing the relevance of the respective process.

## Figures and Tables

**Table 1:** Specimen codes based on 3D printing parameters.

Specimen Code	Printing Parameters		
	Printing Speed (mm/s)	Infill Density (%)	Temperature (°C)
T410	40	100	200
T470	40	75	200
T450	40	50	200
T411	40	100	210
T471	40	75	210
T451	40	50	210
T412	40	100	220
T472	40	75	220
T452	40	50	220
T413	40	100	230
T473	40	75	230
T453	40	50	230
T610	60	100	200
T670	60	75	200
T650	60	50	200
T611	60	100	210
T671	60	75	210
T651	60	50	210
T612	60	100	220
T672	60	75	220
T652	60	50	220
T613	60	100	230
T673	60	75	230
T653	60	50	230
T810	80	100	200
T870	80	75	200
T850	80	50	200
T811	80	100	210
T871	80	75	210
T851	80	50	210
T812	80	100	220
T872	80	75	220
T852	80	50	220
T813	80	100	230
T873	80	75	230
T853	80	50	230

**Table 2:** Results of the tensile test.

Specimen Code	Test Results			
	Modulus of Elasticity (GPa)	Extension (%)	Yield Strength (MPa)	Ultimate Strength (MPa)
T410	3.6	4.4	25.0	28.0
T470	3.0	3.9	27.4	29.5
T450	3.0	4.4	27.8	29.7
T411	3.2	3.9	24.0	26.2
T471	3.0	3.7	26.2	27.3
T451	2.8	5.8	28.8	31.0
T412	2.6	4.7	23.4	24.2
T472	3.6	3.3	27.0	28.5
T452	2.6	5.1	28.0	30.3
T413	4.4	4.6	42.2	45.2
T473	3.4	5.5	35.6	39.4
T453	3.0	6.2	30.4	33.1
T610	3.8	3.4	29.0	30.8
T670	3.0	3.0	22.2	24.8
T650	3.0	3.9	24.6	25.9
T611	3.6	2.9	27.4	29.7
T671	3.0	3.4	24.2	25.9
T651	2.6	4.5	26.2	28.5
T612	3.6	4.8	38.4	42.5
T672	2.8	4.9	32.8	36.0
T652	2.8	4.3	26.2	27.3
T613	3.2	4.3	36.2	41.7
T673	2.8	5.3	38.6	42.1
T653	2.6	6.9	29.2	35.8
T810	3.8	4.3	35.0	39.1
T870	2.6	4.0	23.4	27.3
T850	1.8	4.9	28.8	31.4
T811	3.0	4.5	30.1	32.6
T871	2.6	5.0	29.2	35.8
T851	1.6	5.0	24.0	26.9
T812	2.6	3.6	25.6	28.0
T872	2.6	5.4	30.4	33.3
T852	2.0	6.1	27.2	29.6
T813	2.6	6.6	26.6	34.6
T873	2.2	6.1	33.8	39.0
T853	2.4	6.4	27.2	35.6

**Table 3:** Comparison between tensile test results and simulation results.

Specimen Code	Test Results	Simulation Results		
	UTS (MPa)	UTS (MPa)	Error [%]	UTS in Gap (MPa)
T410	28.0	29.1	3.9	-
T470	29.5	33.1	12.2	56.2
T450	29.7	35.3	18.9	53.7
T411	26.2	27.2	3.8	-
T471	27.3	30.9	13.2	52.0
T451	31.0	36.8	18.7	57.1
T412	24.2	25.2	4.1	-
T472	28.5	34.0	19.3	57.7
T452	30.3	36.0	18.8	54.4
T413	45.2	46.9	3.8	-
T473	39.4	44.6	13.2	71.1
T453	33.1	39.3	18.7	61.0
T610	30.8	32.0	3.9	-
T670	24.8	27.9	12.5	47.3
T650	25.9	30.7	18.5	47.7
T611	29.7	30.9	4.0	-
T671	25.9	27.8	7.3	49.3
T651	28.5	33.8	18.6	52.6
T612	42.5	44.2	4.0	-
T672	36.0	39.8	10.6	68.5
T652	27.3	32.4	18.7	50.3
T613	41.7	43.4	4.1	-
T673	42.1	46.8	11.2	58.2
T653	35.8	42.6	19.0	66.0
T810	39.1	40.7	4.1	-
T870	27.3	30.4	11.4	51.9
T850	31.4	37.2	18.5	57.8
T811	32.6	33.9	4.0	-
T871	35.8	39.6	10.6	68.1
T851	26.9	32.0	19.0	49.7
T812	28.0	29.1	3.9	-
T872	33.3	36.8	10.5	63.4
T852	29.6	35.1	18.6	50.8
T813	34.6	35.9	3.8	-
T873	39.0	43.1	10.5	74.2
T853	35.6	42.3	18.8	65.6

## Data Availability

The original contributions presented in this study are included in the article. Further inquiries can be directed to the corresponding author(s).

## References

[1] Samykano M. (2021). Mechanical Property and Prediction Model for FDM-3D Printed Polylactic Acid (PLA). Arab. J. Sci. Eng..

[2] Gonabadi H., Yadav A., Bull S.J. (2020). The Effect of Processing Parameters on the Mechanical Characteristics of PLA Produced by a 3D FFF Printer. Int. J. Adv. Manuf. Technol..

[3] Tymrak B.M., Kreiger M., Pearce J.M. (2014). Mechanical Properties of Components Fabricated with Open-Source 3-D Printers under Realistic Environmental Conditions. Mater. Des..

[4] Ouhsti M., El Haddadi B., Belhouideg S. (2018). Effect of Printing Parameters on the Mechanical Properties of Parts Fabricated with Open-Source 3D Printers in PLA by Fused Deposition Modeling. Mech. Mech. Eng..

[5] Yeoh C.K., Cheah C.S., Pushpanathan R., Song C.C., Tan M.A., Teh P.L. (2020). Effect of Infill Pattern on Mechanical Properties of 3D Printed PLA and CPLA. IOP Conf. Ser. Mater. Sci. Eng..

[6] Rismalia M., Hidajat S.C., Permana I.G.R., Hadisujoto B., Muslimin M., Triawan F. (2019). Infill Pattern and Density Effects on the Tensile Properties of 3D Printed PLA Material. J. Phys. Conf. Ser..

[7] Ambati S.S., Ambatipudi R. (2022). Effect of Infill Density and Infill Pattern on the Mechanical Properties of 3D Printed PLA Parts. Mater. Today Proc..

[8] Yadav P., Sahai A., Sharma R.S. (2021). Strength and Surface Characteristics of FDM-Based 3D Printed PLA Parts for Multiple Infill Design Patterns. J. Inst. Eng. Ser. C.

[9] Kafshgar A.R., Rostami S., Aliha M.R.M., Berto F. (2021). Optimization of Properties for 3D Printed PLA Material Using Taguchi, ANOVA and Multi-Objective Methodologies. Procedia Struct. Integr..

[10] Wang S., Ma Y., Deng Z., Zhang S., Cai J. (2020). Effects of Fused Deposition Modeling Process Parameters on Tensile, Dynamic Mechanical Properties of 3D Printed Polylactic Acid Materials. Polym. Test.

[11] Gaweł A., Kuciel S. (2020). The Study of Physico-Mechanical Properties of Polylactide Composites with Different Level of Infill Produced by the Fdm Method. Polymers.

[12] Ferreira R.T.L., Amatte I.C., Dutra T.A., Bürger D. (2017). Experimental Characterization and Micrography of 3D Printed PLA and PLA Reinforced with Short Carbon Fibers. Compos. B Eng..

[13] Xu Z., Fostervold R., Razavi N. (2021). Thickness Effect on the Mechanical Behavior of PLA Specimens Fabricated via Fused Deposition Modeling. Procedia Struct. Integr..

[14] Yao T., Ye J., Deng Z., Zhang K., Ma Y., Ouyang H. (2020). Tensile Failure Strength and Separation Angle of FDM 3D Printing PLA Material: Experimental and Theoretical Analyses. Compos. B Eng..

[15] Benwood C., Anstey A., Andrzejewski J., Misra M., Mohanty A.K. (2018). Improving the Impact Strength and Heat Resistance of 3D Printed Models: Structure, Property, and Processing Correlationships during Fused Deposition Modeling (FDM) of Poly(Lactic Acid). ACS Omega.

[16] Gaweł A., Kuciel S., Liber-Kneć A., Mierzwiński D. (2023). Examination of Low-Cyclic Fatigue Tests and Poisson’s Ratio Depending on the Different Infill Density of Polylactide (PLA) Produced by the Fused Deposition Modeling Method. Polymers.

[17] Motoc D.L., Draghicescu H.T., Florea D., Preda I., Ispas N. (2020). Insights into Mechanical and Thermal Properties of Additively Manufactured PLA Samples Triggered by Automotive Industry Demands. Mater. Plast..

[18] Vorkapić M., Mladenović I., Pergal M., Ivanov T., Baltić M. (2022). Optimisation of Tensile Stress of Poly (Lactic Acid) 3D Printed Materials Using Response Surface Methodology. Tribol. Mater..

[19] Hikmat M., Rostam S., Ahmed Y.M. (2021). Investigation of Tensile Property-Based Taguchi Method of PLA Parts Fabricated by FDM 3D Printing Technology. Results Eng..

[20] Tang C., Liu J., Yang Y., Liu Y., Jiang S., Hao W. (2020). Effect of Process Parameters on Mechanical Properties of 3D Printed PLA Lattice Structures. Compos. Part C Open Access.

[21] Akhoundi B., Behravesh A.H. (2019). Effect of Filling Pattern on the Tensile and Flexural Mechanical Properties of FDM 3D Printed Products. Exp. Mech..

[22] Ansari A.A., Kamil M. (2021). Effect of Print Speed and Extrusion Temperature on Properties of 3D Printed PLA Using Fused Deposition Modeling Process. Mater. Today Proc..

[23] Pastor-Artigues M.-M., Roure-Fernández F., Ayneto-Gubert X., Bonada-Bo J., Pérez-Guindal E., Buj-Corral I. (2019). Elastic Asymmetry of PLA Material in FDM-Printed Parts: Considerations Concerning Experimental Characterisation for Use in Numerical Simulations. Materials.

[24] Yu Z., Gao Y., Jiang J., Gu H., Lv S., Ni H., Wang X., Jia C. (2019). Study on Effects of FDM 3D Printing Parameters on Mechanical Properties of Polylactic Acid. IOP Conf. Ser. Mater. Sci. Eng..

[25] Pachauri S., Gupta N., Gupta A. (2023). Influence of 3D Printing Process Parameters on the Mechanical Properties of Polylactic Acid (PLA) Printed with Fused Filament Fabrication: Experimental and Statistical Analysis. Int. J. Interact. Des. Manuf. (IJIDeM).

[26] Kołodziej A., Żur P., Borek W. (2019). Influence of 3D-Printing Parameters on Mechanical Properties of PLA Defined in the Static Bending Test. Eur. J. Eng. Sci. Technol..

[27] Grasso M., Azzouz L., Ruiz-Hincapie P., Zarrelli M., Ren G. (2018). Effect of Temperature on the Mechanical Properties of 3D-Printed PLA Tensile Specimens. Rapid Prototyp. J..

[28] Marșavina L., Vălean C., Mărghitaș M., Linul E., Razavi N., Berto F., Brighenti R. (2022). Effect of the Manufacturing Parameters on the Tensile and Fracture Properties of FDM 3D-Printed PLA Specimens. Eng. Fract. Mech..

[29] Tanveer M.Q., Haleem A., Suhaib M. (2019). Effect of Variable Infill Density on Mechanical Behaviour of 3-D Printed PLA Specimen: An Experimental Investigation. SN Appl. Sci..

